# Mega-phylogeny approach for comparative biology: an alternative to supertree and supermatrix approaches

**DOI:** 10.1186/1471-2148-9-37

**Published:** 2009-02-11

**Authors:** Stephen A Smith, Jeremy M Beaulieu, Michael J Donoghue

**Affiliations:** 1 National Evolutionary Synthesis Center, 2024 W Main St A200, Durham, NC 27705, USA; 2 Department of Ecology and Evolutionary Biology, Yale University, PO Box 208105, New Haven, CT 06520, USA

## Abstract

**Background:**

Biology has increasingly recognized the necessity to build and utilize larger phylogenies to address broad evolutionary questions. Large phylogenies have facilitated the discovery of differential rates of molecular evolution between trees and herbs. They have helped us understand the diversification patterns of mammals as well as the patterns of seed evolution. In addition to these broad evolutionary questions there is increasing awareness of the importance of large phylogenies for addressing conservation issues such as biodiversity hotspots and response to global change. Two major classes of methods have been employed to accomplish the large tree-building task: supertrees and supermatrices. Although these methods are continually being developed, they have yet to be made fully accessible to comparative biologists making extremely large trees rare.

**Results:**

Here we describe and demonstrate a modified supermatrix method termed mega-phylogeny that uses databased sequences as well as taxonomic hierarchies to make extremely large trees with denser matrices than supermatrices. The two major challenges facing large-scale supermatrix phylogenetics are assembling large data matrices from databases and reconstructing trees from those datasets. The mega-phylogeny approach addresses the former as the latter is accomplished by employing recently developed methods that have greatly reduced the run time of large phylogeny construction. We present an algorithm that requires relatively little human intervention. The implemented algorithm is demonstrated with a dataset and phylogeny for Asterales (within Campanulidae) containing 4954 species and 12,033 sites and an *rbcL *matrix for green plants (Viridiplantae) with 13,533 species and 1,401 sites.

**Conclusion:**

By examining much larger phylogenies, patterns emerge that were otherwise unseen. The phylogeny of Viridiplantae successfully reconstructs major relationships of vascular plants that previously required many more genes. These demonstrations underscore the importance of using large phylogenies to uncover important evolutionary patterns and we present a fast and simple method for constructing these phylogenies.

## Background

All species on Earth – current estimates exceed 1.8 million – are related through common ancestors in the evolutionary Tree of Life. The construction of this phylogeny is a major endeavor for biology and largely now depends on the unprecedented growth of molecular sequence data available in public databases. Efforts focused on single clades, whole genome sequencing, genomic library construction (ESTs, BACs), and large collaborative efforts, such as NSF's Assembling the Tree of Life project, are contributing to the fast-paced growth of public databases, with more than 92 million sequences stored in the current release of GenBank (release 167). Current efforts to infer really large phylogenetic trees center on data combination using so-called supertree [e.g., [1]] and supermatrix methods [e.g., [2-4]] as opposed to using a single gene (or multiple genes) sampled very widely across taxa [e.g., [5,6]]. For example, recent large-scale database-enabled phylogenetic analyses employing these approaches have shed light on the radiation and early evolution of mammals [1], and the phylogenetic diversity of Bacteria [3]. Recent advances in phylogenetic tree-building methods have provided the necessary first steps in approaching the problem of producing large and comprehensive phylogenetic trees [7-9]. However, assembling large datasets from databases remains a critical problem upstream of the tree-building process.

Supertree methods compile many source trees with partially overlapping taxa into a single comprehensive tree [10,11]. Generally, each source topology is converted into a data matrix and combined with other topological matrices. Many different algorithms exist for creating the final supertree including MRP (matrix representation with parsimony; [12,13]), MRF (matrix representation with flipping; [14]), MinCut [15], and modified MinCut [16]. Although straightforward, supertree methods are not without their limitations, including problems related to data independence (same data can contribute to more than one source tree), "signal enhancement" ([11] novel relationships in supertrees contradicting one or several source trees), and the assessment of uncertainty and confidence in relationships [17]. In addition, supertrees are strictly topological, thus requiring sequence data to obtain useful branch lengths [1]. Most importantly however, supertrees do not directly rely on the primary data for tree inference, making novel topologies suspect. Perhaps due to these limitations, and despite active development of methodologies [e.g., [1]], few large supertrees for diverse groups have been successfully constructed (but see [18,19]).

Supermatrix methods, on the other hand, are directly inferred from the sequence data through the construction of a large multiple sequence alignment for simultaneous analysis of the final data-matrix [20]. Given the fact that few genes are sampled very completely across many taxa, supermatrix methods often sacrifice completeness in the interest of size. In fact, one of the largest supermatrices, with >2000 tips, had 95% missing data [4]. Other supermatrix analyses have focused on the number of gene regions and not on the number of species [2,3]. The construction of a large supermatrix involves a number of computationally challenging steps including, but not limited to, database operations, BLAST comparisons, sequence clustering, multiple sequence alignment, and combining data sets. An exhaustive discussion of these steps is presented elsewhere [4], but each will be briefly touched upon as it relates to the approach presented here. Typically, sequences have been deposited in a database and all-by-all sequence comparisons with BLAST are conducted to assemble sequence clusters based on similarity. Methods for this step include agglomerative procedures, like single linkage clustering (e.g. blastclust) and stochastic methods (e.g. Tribe-MCL; [21]). Clustered sequences are then submitted to multiple sequence alignment (MSA). There are a host of other procedures that can be conducted once multiple sequence alignments are produced, especially related to identifying sequence orthology. Multi-locus datasets are created from individual alignments that do not have "too many" missing entries using a bipartite graph of taxa and loci and combining with bicliques or quasi-bicliques [22,23].

Each step described above is computationally difficult and rarely has been discussed in the context of what might be optimal for the final goal of tree construction. Despite the computational difficulties and potential shortcomings of specific steps in their construction, supermatrix methods allow for simultaneous data analysis. Also, unlike supertrees, they do not suffer from data independence or "signal enhancement" problems, and, at least in principle, confidence can be assessed using standard bootstrapping approaches. However, problems related to missing data and assessing the quality of the trees produced persists. Tools addressing certain steps of supermatrix construction are beginning to become available (e.g., Phylota; [24]) and some notable large trees have been successfully produced [4]. However, tools for constructing supermatrices are not readily available for comparative biologists, and rather few large matrices for specific clades have been successfully analyzed. Nevertheless, supermatrix methods have made enormous strides forward and recent discussions have begun to center on methods that combine elements of both supertree and supermatrix approaches [e.g. [17,25,26]].

The method introduced here, to which we refer to as a "mega-phylogeny", is most similar to supermatrix methods, but differs from previous methods used to create large matrices in a number of ways. The mega-phylogeny method relies on the user identifying the gene regions of interest by presenting actual examples of the gene region and the breadth of molecular diversity of that gene within the clade of interest. Also, the mega-phylogeny method employs profile alignments to combine alignments of orthologous gene regions that would either be poorly aligned if done across a broad taxonomic group or would be broken up by clustering analyses. The mega-phylogeny approach can quickly create enormous phylogenetic matrices as more data from the same gene may be used, and the problems associated with sequence saturation are specifically attenuated. The first demonstration of this method [27] produced phylogenies for plant clades from 366 species and 11,374 sites (Dipsacales) to 4657 species and 22,391 sites (Commelinidae).

Here, we describe this new approach and its current implementation. We also present two example phylogenies for two plant clades created using our method: an Asterales phylogeny containing 4954 species and five gene regions and an *rbcL *phylogeny of green plants (Viridiplantae) comprising more than 13,533 species.

## Methods

### Implemented Pipeline

The basic steps for a mega-phylogeny include (1) designating the clade of interest, (2) identifying the gene region(s) of interest, (3) recording the extent of molecular diversity of the gene region in the clade of interest, (4) recording the threshold of coverage and identity to be used for orthology tests, (5) narrowing the possible sequences with a very broad term search [optional], (6) remove all potential sequences that are not members of the clade of interest, (7) testing orthology by BLASTing each potential sequence to each gene region identified for the breadth and removing those sequences that differ by more than the established threshold, (8) identifying sequences that should be reverse complemented, (9) removing sequences for duplicate taxon names, keeping the sequence with the best coverage and identity, and (10) test for saturation. If the sequences are saturated, subdivide them using the next available subclade and perform additional tests of saturation (step 10). Finally, once all of the sequences are in an alignment or exist as singletons (i.e. are not found to be contained in any subdivisions), profile each alignment to a master alignment. This can be repeated an arbitrary number of times for each gene region of interest. If multiple gene regions are used, these are then concatenated into a large matrix and the phylogeny inferred.

We implemented this pipeline in Python (vers. 2.5) with the BioPython (vers. 1.48) module and using the BioSQL (vers. 1.0.1) database schema. Each mega-phylogeny matrix assembly analysis presented here was run on a Linux laptop with 1 GB RAM and a 2.4 Ghz dual-core processor. The phylogenetic analyses were conducted on an eight-way SMP Linux computer with 2.4 Ghz processors and 32 GB of RAM using RAxML (vers. 7.0.4; [8]). The steps that are novel for matrix assembly are described briefly below.

### Orthology

Determining whether sequences are orthologous is a challenge for large tree construction. Supermatrix methods have attempted to overcome this problem by identifying orthologous sets of sequences using clustering techniques [2,4], but these can be time consuming and are typically not developed with the goal of large phylogeny assembly [e.g., [28]]. Here, we determine orthologous sequences using designated sequences representing the breadth of variation observed in the gene region of interest across the clade of interest. We BLAST all of the potential sequences from the database against these designated sequences and other potential sequences that are determined to match with a certain threshold (i.e. according to both coverage and identity). At this stage, reverse complements are corrected by determining which direction best matches the designated regions of interest. Instead of N × N comparisons between each potentially useful sequence, only N × n comparisons are necessary, where n is the number of example sequences used to represent the region. This dramatically shortened the run time of the algorithm as well as generally produced denser matrices.

### Profile alignments

One major problem for large matrices using broadly sampled sequences or smaller matrices with quickly evolving sequences is that multiple sequence alignments become more challenging as sequences become more divergent [28,29]. Almost all multiple sequence alignment algorithms build a phylogeny during the estimation procedures [30]. The phylogenies built for multiple sequence alignment are often based on model-corrected or raw pair-wise distances. These methods are susceptible to problems with saturation (i.e. multiple mutations at the same site for the same organism) and are therefore much less accurate for large and broadly sampled alignments that are likely to contain very distantly related sequences. The quality of multiple sequence alignments can have a dramatic impact on the accuracy of the phylogenies produced [e.g., [31-33]]. As a result, other supermatrix methods sidestep multiple sequence alignment by employing clustering techniques to determine "alignable regions." Such clustering techniques have allowed for the assembly of very large matrices of sufficiently similar sequences [2,4,34]. This approach can be dramatically affected by the parameters used during clustering, sometimes resulting in multiple informative clusters for slower gene regions (e.g. two large *rbcL *clusters for Ericales in Phylota).

We combine the analysis of sequence saturation with recent advances in multiple profile-to-profile alignment methodology. A profile alignment is an algorithmic approach to identifying structural elements that are highly conserved between different alignments [34-36]. To accomplish this, separate alignments are aligned together while preserving the columns in the individual alignments. Newer profile alignment programs allow for more flexibility in profile alignment procedures (e.g. MAFFT; [37]). In our case, we separate sequences into subgroups of aligned sequences based on the degree of sequence saturation. For example, if the algorithm determines that the most inclusive group of sequences is saturated, then the group is broken up into less inclusive groups using the next level in the taxonomic hierarchy. In a Linnaean taxonomic system, if an "order" is found to be saturated, it would be broken into "families". Each smaller subset of sequences is then re-aligned and the saturation reassessed. This process continues iteratively to less inclusive groups until sequences no longer appear saturated and these alignments are then stored. We note, however, that the taxonomic groups used in this procedure need not correspond to ranks in the Linnaean hierarchy, but should simply be hierarchically nested (as in the NCBI taxonomy). Grouping using a rank-free classification (PhyloCode; [38]) could be used and will be possible once a database of phylogenetic names is implemented and usable. After every sequence has been either placed in an alignment or placed as a "singleton," the individual alignments are then profiled to a larger alignment. The order of the profiling can be random, optimized to find the best order, or aided by a hierarchical "guide" tree (e.g. first aligning more closely related matrices). Currently, we employ highly conservative guide trees based on published studies to carry out profile alignments.

### Assessing saturation

We introduce a simple method based on dispersion statistics to rapidly detect saturation across a set of sequence data. Dispersion (an indicator of spread) is assessed on the one-dimensional Euclidean distance between the raw pair-wise sequence distances and those corrected according to a Jukes-Cantor model of molecular substitution. A one-dimensional Euclidean distance is the absolute difference between two points. Our measure of dispersion is based on the median and is commonly referred to as the median absolute deviation (MAD) and given by

MAD = 1.4826 × Med (| x_i _- Med (x)|),

where the median is estimated from the residual variation about the median of all pair-wise Euclidean distances. The constant 1.4826 is used to make MAD consistent for the standard deviation [39]. Thus, in our use, the larger the MAD the larger the overall spread in the Euclidean distances – that is, above a certain value the assumed nucleotide substitution model is no longer adequately accounting for the rate variation exhibited by pair-wise distances among species.

We performed a simple simulation study to explore the behavior of MAD. First, we wanted to determine a threshold for subdividing sequences into smaller alignments. In addition, we wanted to compare MAD with alternative measures of dispersion based on the sample mean (i.e. mean square error, MSE; root mean square, RMSE). Sequence data were simulated across randomly constructed 20- and 100-tip phylogenies. Different rates of molecular evolution were simulated by incrementally scaling the total tree length by a factor of 0.10, starting from 0.10 and stopping at 2.0. All molecular simulations were carried out using Seq-gen (Ver 1.3.2; [40]).

**Figure 1:**
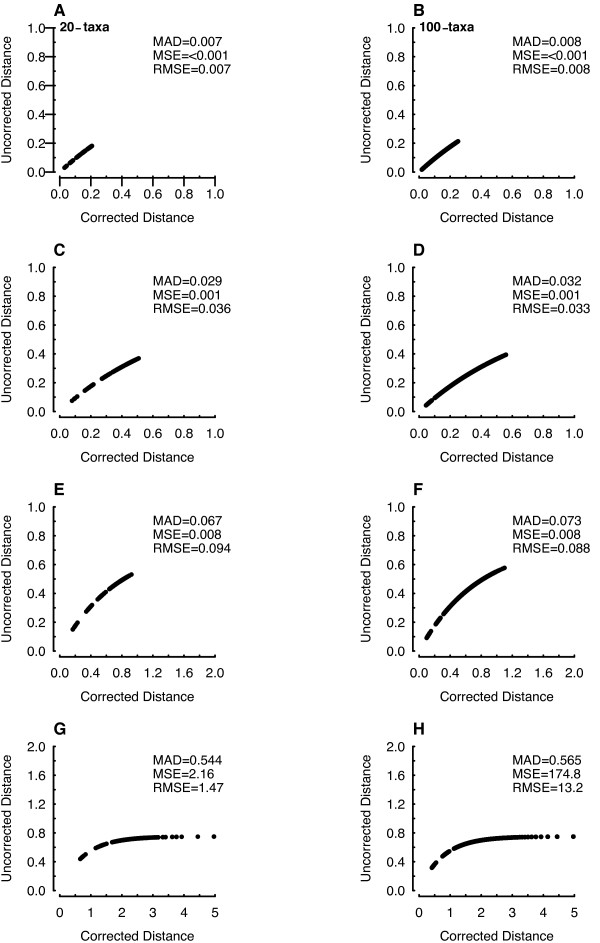
**Simulation exploring the behavior of MAD in relation to alternative measures of dispersion**. Each panel is a simulation of sequence data on a balanced phylogeny of 20-(A, C, E, and G) and 100-tips (B, D, F, and H). A and B total tree length scaled to 0.10. C and D total tree length scaled to 0.25. E and F total tree length scaled to 0.50. G and H total tree length scaled to 2.00. Saturation was assessed by descriptors of dispersion on the one-dimensional Euclidean distance between the raw pair-wise sequence distances (uncorrected distance) and those corrected according to a Jukes-Cantor model of molecular substitution (corrected distance). Our simulations demonstrated that the use of the non-parametric median absolute deviation (MAD) had several advantages of detecting saturation over alternative measures of dispersion based on the sample mean (i.e. mean square error, MSE; root mean square, RMSE).

The results from these simulations clearly highlight the utility of MAD. First, unlike MSE or RMSE, MAD does not require an underlying Gaussian distribution, which is useful as the distribution of Euclidean distances becomes skewed as the degree of sequence divergence increases. A second advantage, and perhaps the most critical, is that MAD appears stable when sequence divergence is unrealistically high (e.g. tree length scaled by a factor of 2; Figure 1). This situation is also analogous to the presence of outliers that have well-known influences on dispersion statistics based on the mean. Because MAD does not require an explicit distribution and is the 50^th ^percentile of the residual variation, it has the inherent property of being robust to outliers (Figure 1G, H). Finally, our simulations indicate that a MAD exceeding ~0.01 provided a conservative indication of a saturation level necessitating a profile alignment scheme.

## Results

### Asterales

Nearly 10% of all angiosperms are contained within the Asterales; a clade that is mainly comprised of 12 recognized families with a majority of the diversity being attributed to just two families, Asteraceae (e.g. sunflowers, thistles) and Campanulaceae (e.g. *Lobelia *and relatives). The monophyly of Asterales is well supported despite uncertainty in its position within the more inclusive Campanulidae clade [41]. There are roughly 5200 species of Asterales represented in GenBank, or roughly 20% of the entire clade. However, aside from studies of carefully selected exemplar taxa representing major lineages of Asterales [41-46], a comprehensive phylogeny has never been produced for this clade. Here we apply our mega-phylogeny approach to the Asterales to reconstruct the most complete phylogeny of the group to date.

Our Asterales sequence matrix was comprised of *rbcL*, *mat*K, *trnL-F*, *trnK*, *ndhF*, and ITS. The combined matrix of 12,033 sites was comprised of 90.959% gaps or missing sequence. However, the individual gene regions were more variable in gap or missing sequence composition: 98.043% in ETS, 36.348% in ITS, 98.188% in *matK*, 90.338% in *ndhF*, 92.597% in *rbcL*, 98.002% in *trnK*, and 81.445% in *trnL-F*. Of the five gene regions sampled, ITS was the best represented taxonomically (with 4242 species) and was the only region identified by our procedure as requiring profile-to-profile alignments. The MAD score indicated that the degree of ITS saturation varied among groups, but within-group alignments were never carried out above the traditional "tribal" level. This resulted in 180 separate within-group alignment files of differing hierarchical level.

As an efficient means to direct the profile-to-profile alignments, we assembled a "guide" tree by compiling and grafting together published phylogenies (*sensu *[47]). Briefly, we first obtained a backbone phylogeny from Winkworth et al., [41] and Lundberg and Bremer [44] for the major lineages of Asterales. We then grafted trees based on more focused studies into the backbone tree. We started this process with the most inclusive clade and proceeding "inwards", adding more and more detailed analyses of included clades. Our final grafted tree was pruned down to correspond to the 180-alignment files output from our saturation analysis (see Additional file 1). This guide tree was then traversed in a post-order fashion, performing profile-to-profile alignments starting at the "terminals" and working recursively back to the root. The phylogeny was then inferred using RAxML (vers. 7.0.4; [8]), partitioning for each gene region using the GTR+GAMMA model of rate substitution.

**Figure 2:**
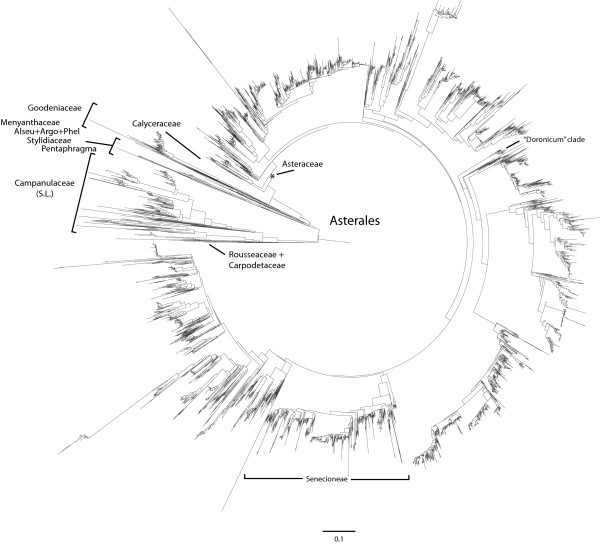
**Maximum-likelihood phylogeny for 4954 species of Asterales**. The data matrix was constructed using the mega-phylogeny method and includes DNA sequences for five genes: *rbcL, matK, trnL-F, ndhF*, and ITS. Each of the 12 major families of Asterales is labeled. We also note the placement of the "Doronicum" clade in relation to the tribe Senecioneae; although we assumed a sister relationship *a priori*, the phylogenetic analysis overruled this assumption, indicating that the two clades may be more distantly related. Pentaphragma, Pentaphragmataceae; Alseu, Alseuosmiaceae; Argo, Argophyllaceae; Phel, Phellinaceae.

Our final phylogeny includes 4954 tips with the branching within and among "families" being mostly consistent with previously published results (Figure 2). One exception concerns the early branching lineages of Asterales, involving the placement of Rousseaceae+Carpodetaceae, Campanulaceae, and Pentaphragmataceae. The current consensus recognizes a basal trichotomy among these three clades ([48]; but see [41,44]). Our analysis recovered Rousseaceae+Carpodetaceae as the sister groups of all other Asterales, within which Campanulaceae (including Lobeliaceae) is sister to the rest (Figure 2). This result has been recovered before [44], but other studies have suggested a basal split between Rousseaceae+Carpodetaceae plus Campanulaceae [41,44] and all the rest. Our analysis shows Pentaphragmataceae as sister to a clade comprising Stylidiaceae, Alseuosmiaceae, Argophyllaceae, Phellinaceae, Menyanthaceae, Goodeniaceae, Calyceraceae, and Asteraceae (Figure 2). A recent combined analysis of chloroplast and nuclear genes found strong support for this relationship [41].

Relationships within Asteraceae coincide well the subfamilial classification of Panero and Funk [46]. However, we note that our phylogeny does not support the monophyly of Wunderlichioideae, Stifftioideae, Mutisioideae, or Gochnatioideae (*sensu *[46]). Instead we found these groups to be broken into smaller successive sister clades to the rest of Asteraceae.

Several relationships, within major clades, are worth noting as they highlight the utility of our method. Based on the NCBI taxonomy, the profile-alignment portion of our algorithm assumed that *Campanula *was monophyletic. However, the MAD score detected extreme sequence variation that required profile alignments among species. This variation is an indication of extreme molecular differentiation, and in the case of *Campanula*, paraphyly, which is consistent with more focused systematic studies of Campanulaceae [49,50]. In several cases we also assumed sister relationships where the primary literature suggested low nodal support. For example, we profiled the genus *Doronicum *and the tribe Senecioneae as sister clades within the more inclusive Asteroideae, though there is generally low confidence in this hypothesis [51]. The resulting tree showed these two clades to be more distantly related, as *Doronicum *is placed near the early branching lineages of Asteroideae (Figure 2). Taken together, these results show that even though we assume some phylogenetic relationships at the outset in doing the profile alignments, our results need not recover the same relationship within the final phylogenetic tree.

### Green plants

The green plants (Viridiplantae) contain more than 350,000 species including green algae, liverworts, mosses, ferns, and seed plants, including the flowering plants. The early branches of the entire clade and of each major group of green plants have attracted extensive molecular work [52-55]. Two large clades of living green plants are supported: Streptophyta and Chlorophyta [52]. Charophytes (stonewarts) have been supported as the sister group to land plants based on the inclusion of six genes [53,55]. Despite the large number of molecular studies that have focused on deep relationships within green plants, few studies with very large numbers of taxa have been conducted.

Here, we create a mega-phylogeny of the chloroplast ribulose-bisphosphate carboxylase (*rbcL*) gene for all green plants. This well sampled gene has been extensively examined in smaller studies throughout plants, especially in flowering plants [beginning with 5]. Despite the widespread use of *rbcL*, the addition of other genes has generally been necessary to confidently reconstruct many relationships. Here, our goal was to construct the largest *rbcL *phylogeny for green plants while simultaneously accommodating saturation across the alignment.

Over 16,000 *rbcL *sequences were found to be orthologous to the designated sequences sampled throughout flowering plants. Our final matrix with duplicate taxa removed consisted of 13,533 tips and 1401 nucleotide sites with 4.6238% of the matrix consisting of gaps. Our saturation analysis recognized 15 separate aligned subgroupings: Chlorophyta (486 sp.), Zygnesnophyceae (131 sp.), Coleochaetophyceae (21 sp.), Charophyceae (34 sp.), Marchantiophyta (462 sp.), Bryophyta (570 sp.), Anthocerotophyta (56 sp.), Lycopodiopsida (48 sp.), Isoetopsida (101 sp.), Equisetophyta (18 sp.), Marattiopsida (59 sp.), Ophioglossopsida (29 sp.), Filicopsida (1624 sp.), Psilotophyta (3 sp.), and Spermatophyta (9900). These aligned subgroups were combined using profile alignments across a guide tree based on Donoghue [56] and Cantino et al. [38] (see additional file 2). The phylogeny was constructed using RAxML (vers. 7.0.4; [8]) using the GTR+GAMMA model of rate substitution.

**Figure 3:**
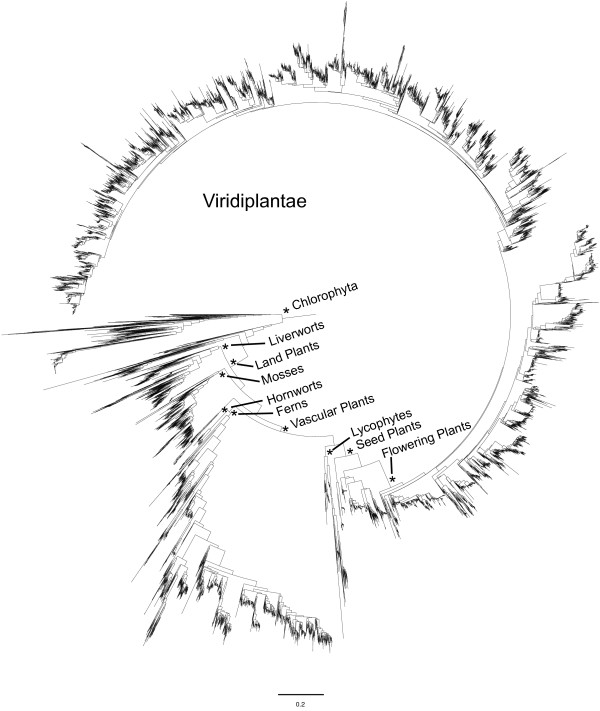
**Maximum-likelihood phylogeny for 13,533 species of green plants based on *rbcL *DNA sequences**. The data matrix was constructed using the mega-phylogeny method; major clades are labeled and denoted with a star.

A recent analysis by Qiu et al. [55] compiled the most comprehensive dataset to date, using six genes and 193 species to resolve relationships of the four major land plant lineages: liverworts, hornworts, mosses, and vascular plants. They recover, with strong support, a resolution of successive sister clades starting with liverworts, then mosses, then hornworts, and vascular plants. Our maximum-likelihood tree of more than 13,000 species recovers this same relationship with the use of *rbcL *alone (Figure 3). However, within vascular plants, our trees differ in the placement of lycophytes. In the Qiu et al. [56] tree, monilophytes are more closely related to seed plants than lycophytes and these relationships are well established based on other evidence (e.g., morphology [57], gene order and gene losses [58,59]). We find the lycophytes to be more closely related to seed plants, which is likely to be mistaken and reflects an artifact in the evolution of *rbcL*.

Our much larger phylogeny resolves some relationships by including more data in the form of more species instead of more genes. This has been documented previously but has rarely been tested on such a large scale [60,61]. With the inclusion of more taxa other broad evolutionary patterns emerge [cf. [27]]. For example, in this case, the ferns appear to have faster rates of evolution than the other vascular plants. Further study is required to quantify this pattern and its important, as the timing and rate of evolution of ferns has been interpreted in light of angiosperm evolution [62]. With more taxa sampled, rate heterogeneity can become more apparent, raising an important issue about the possible effects of clade-specific rates on divergence-time estimates [27]. Unfortunately, accurate estimates of divergence times using tens of thousands of species remain impractical.

Another important result is that *rbcL *appears to be saturated across green plants. That is, despite the conservative nature of this coding region, when looking very broadly there are likely to be multiple mutations at sites throughout the gene causing either less accurate multiple sequence alignments or causing clustering methods to break up the matrix into smaller sections. Broad analyses of green plants will need to take this into account. Our analysis also demonstrates the limitations of conventional computers for analyzing large phylogenies. The matrix manipulation, tree construction, and tree rerooting required at least 8 GB of memory and were conducted on an 8 CPU machine. To build even larger matrices, more memory and faster machines will be essential.

## Discussion and conclusion

The examples presented here demonstrate the utility of our strategy for building large phylogenetic trees. The mega-phylogeny method is capable of producing large and somewhat denser phylogenetic matrices with the addition of human intervention in the selection of gene regions. These matrices can be a partitioned multi-locus dataset, as in the Asterales example, or a single-locus analysis of tens of thousands of terminals, as in the green plants. The size is limited only by computing power. Also, our examples illustrate how well sampled regions (such as ITS) that may be evolving too fast for traditional multiple sequence alignment may be included in broad phylogenetic analyses. Our mega-phylogeny approach also demonstrates that the addition of many more taxa can help resolve relationships where, traditionally, more genes would be required. A direct comparison of our mega-phylogeny method to trees constructed from supermatrix methods is difficult as the two approaches have somewhat different goals. Supermatrix methods can, as implemented by McMahon and Sanderson [[4], also see [2,3]], attempt to make the largest matrix from a database of sequences without specifically identifying particular regions of interest. The mega-phylogeny approach attempts to make the matrix with the largest number of taxa for any clade given the gene regions pre-identified as being of interest. This allows for the creation of somewhat denser matrices, faster. Because fewer gene regions would, in general, be used in the mega-phylogeny approach, partitioned likelihood analyses can be employed more easily with shorter run times. At the moment, there is no standard software for supermatrix methods that could be benchmarked against the mega-phylogeny approach.

Our mega-phylogeny method will perhaps be most useful for comparative biology. In recent years there is an emerging interest in compiling broad-scale datasets to identify general patterns and test specific hypotheses using a phylogenetic framework. For example, new and interesting patterns have emerged in topics ranging from molecular rates [27] to ecophysiology [63-65] to biodiversity [66] and ecosystem processes [67]. However, the level of resolution in the underlying phylogeny has limited many of these studies with many being constructed from multiple literature-based trees (e.g. Phylomatic; [68]). Our method provides a means to construct large phylogenies from primary data, which we hope will facilitate more sophisticated and robust comparative analyses. This has been demonstrated with rate heterogeneity in plants [27]. The limiting factor may soon become the ability of software for various comparative analyses to handle mega-phylogenies.

### Modularity

The mega-phylogeny method is inherently modular, making each step easily extensible. For example, instead of using BLAST comparisons for sequence orthology tests, another test could be used. In fact, a modified clustering method, as is typically found in supermatrix construction, could be utilized. Additionally, instead of the MAD measurement for saturation, other measures could be devised. Concomitantly, any number of different taxonomic databases can be used when saturation is detected. We have relied on the NCBI hierarchical taxonomy, but increased precision might eventually be obtained by using a system containing many additional levels. The modularity of the mega-phylogeny approach encourages its longevity when better methods and approaches become available to address specific procedures underlying mega-phylogeny matrix creation.

Modularity is especially important with respect to the guide trees involved in profile alignment, where the results from different guide trees (or no guide tree) can be compared. For example, there may be a published study of broadly sampled taxa included in the clade of interest for the mega-phylogeny approach. A profile alignment using this tree could easily be compared to one that consists only of basal polytomies, which will be profiled randomly. The use of guide trees for this step highlights the need for available definitive bifurcating trees for profile alignments, especially broadly sampled trees. From this perspective, the compilation of large-scale trees from published phylogenies (e.g., available on TreeBASE, ) becomes a highly relevant endeavor, not only from the standpoint of the initial guide tree but also as a basis for the comparison of results. Important differences could then highlight areas in special need of attention. For example, further attention is needed to the signal in *rbcL *that places lycophytes with seed plants.

### Potential pitfalls

A key element of our mega-phylogeny method is its reliance on prior knowledge of phylogenetic relationships when performing profile-to-profile alignments. We assume that each group being aligned is monophyletic, which is potentially a problem once saturation is detected and less inclusive multiple sequence alignments are employed. However, despite such assumptions, our saturation analysis using the MAD statistic is not irreversibly susceptible to outliers and can detect extreme variation when, for example, it is not monophyletic as demonstrated with *Campanula *within our Asterales matrix. In this case, the MAD score suggested that the assumption of monophyly for *Campanula *was violated, and it emerged as paraphyletic in the final tree. Even though the assumption of monophyly is a potential problem, it is not always detrimental. Further work is needed to explore the sensitivity of the results to such assumptions. In the meantime, the approach highlights the need for taxonomic databases to most accurately reflect current best knowledge of phylogenetic relationships.

Various problems identified in supermatrix construction may also pertain to the mega-phylogeny method. For example, there are problems with database-enabled phylogenetics that are hard or impossible to avoid, such as misidentification or mislabeling in GenBank [4]. Sequence orthology tests can help identify such problems, however outliers are likely to still cause difficulties in some matrices. Additionally, there are potential problems with "rogue taxa" that can lower resolution and support throughout the tree. However, the problem of rogue taxa continues to also be a problem for supermatrices and therefore the development of solutions will likely benefit both methods.

### Extensions

It may be possible to incorporate diversity estimates for each taxonomic group, such that large clades represented by single (or a few) species for a particular gene could be excluded. This would likely reduce problems associated with rogue taxa. Although this information is not currently readily available, its inclusion could greatly increase the efficacy of the mega-phylogeny approach.

Our method can also be extended to deal with the problem of sequence outliers. Unfortunately, the size of the matrices that can be constructed makes checking for outliers by hand impractical. But saturation statistics could be extended to identify these outliers in the individual gene regions. Although the orthology tests and reverse complement procedures identify the vast majority of problematic sequences, the MAD statistic has the potential to cleanse the datasets further, allowing for almost complete automation of large tree construction.

Finally, the mega-phylogeny procedure can be parallelized. Many of the procedures related to sequence-to-sequence comparisons (e.g., orthology tests, reverse complements) can be easily distributed on multiple CPU's or computers. This is also true of some of the multiple sequence alignment calculations. Parallelizing these procedures would yield even faster the mega-phylogenic analyses.

## Authors' contributions

SAS developed the approach and conducted the analyses. JMB developed the saturation tests. SAS, JMB, and MJD wrote and edited the manuscript.

## Supplementary Material

Additional file 1 **Figure S1.** The final assembled "guide" tree used to direct the profile alignments across Asterales. Each "terminal" represents the name of the name of the 180-alignment files output from our saturation analysis. We assembled a "guide" tree representing the relationships among the alignment files by compiling and grafting together published phylogenies. This guide tree was then traversed in a post-order manner, performing each profile-to-profile alignment working recursively back to the root. Click here for file

Additional file 2 **The "guide" tree used to direct the profile alignments across green plants (Viridiplantae).** Similar to Fig S1, each terminal in the tree represents a separate alignment file output from our saturation analysis. Due to the large uncertainty at several nodes, members of a polytomy were profile aligned to find the best order. Click here for file
