# Supplementary figures and images for: Mega-phylogeny approach for comparative biology: an alternative to supertree and supermatrix approaches
Source: BMC Evol Biol. 2009 Feb 11;9:37. doi: 10.1186/1471-2148-9-37 (PMC2645364; doi:10.1186/1471-2148-9-37)

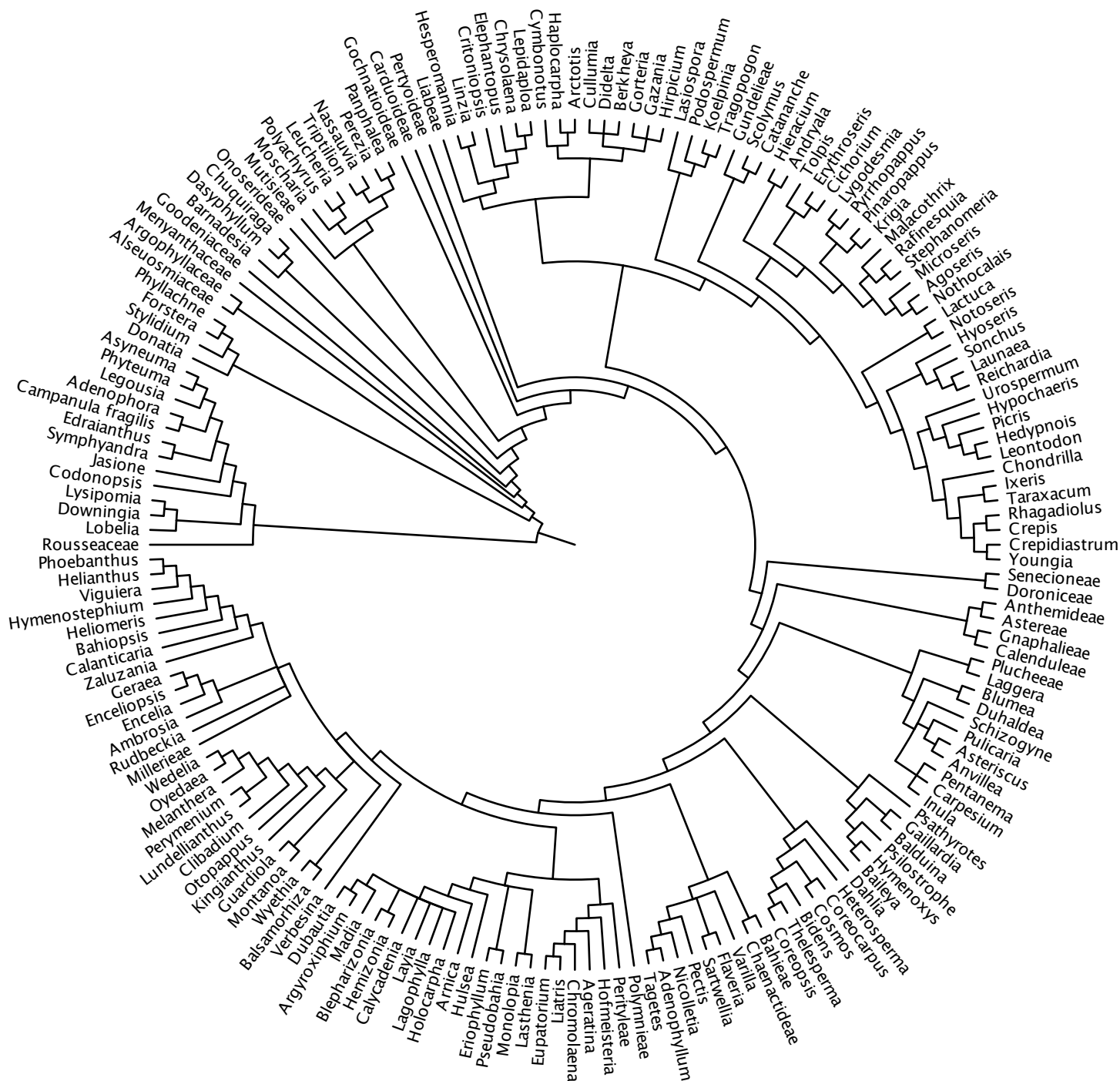

Supplement: Additional file 1 — Figure S1. The final assembled "guide" tree used to direct the profile alignments across Asterales. Each "terminal" represents the name of the name of the 180-alignment files output from our saturation analysis. We assembled a "guide" tree representing the relationships among the alignment files by compiling and grafting together published phylogenies. This guide tree was then traversed in a post-order manner, performing each profile-to-profile alignment working recursively back to the root. [file 1471-2148-9-37-S1.pdf]

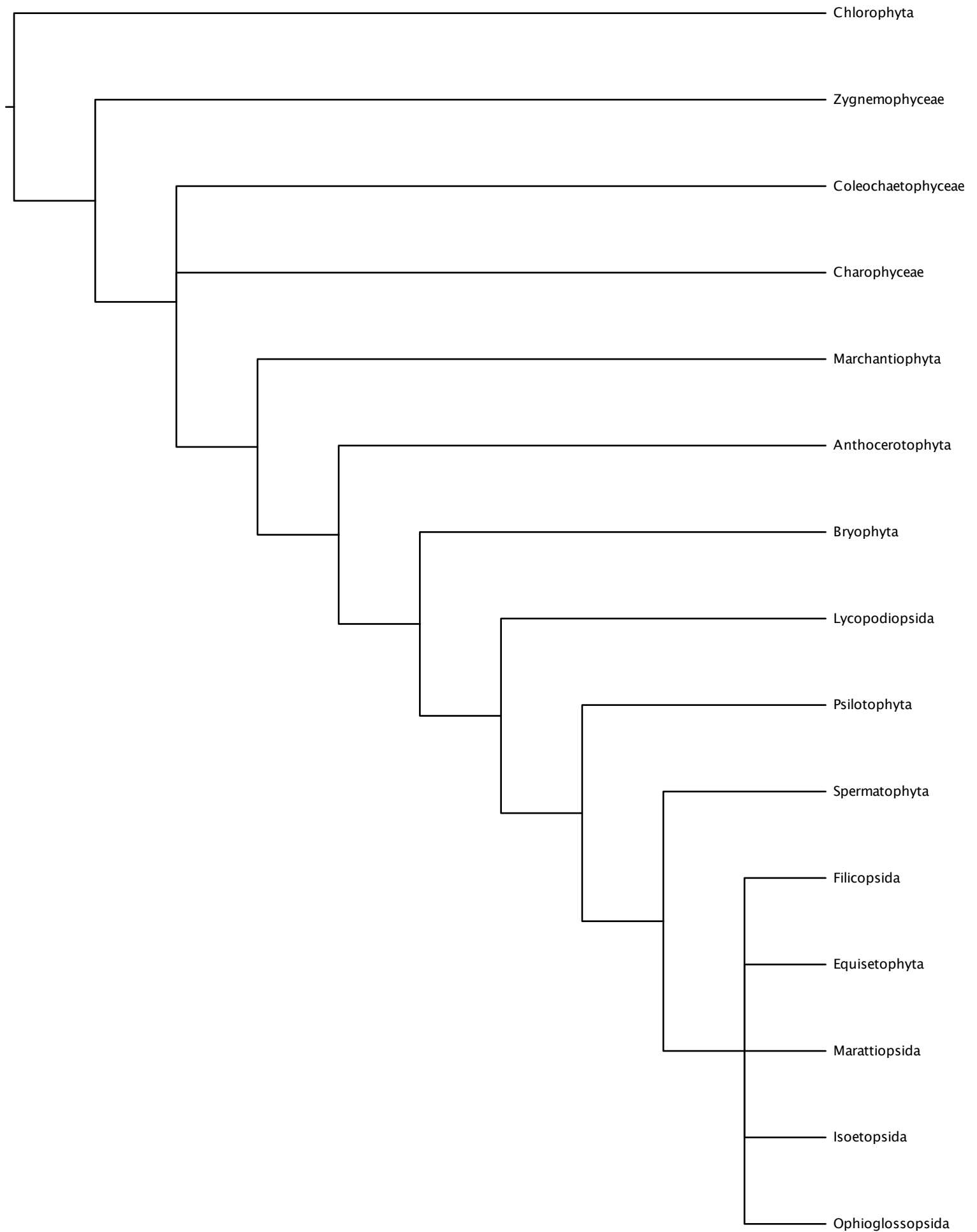

Supplement: Additional file 2 — The "guide" tree used to direct the profile alignments across green plants (Viridiplantae). Similar to Fig S1, each terminal in the tree represents a separate alignment file output from our saturation analysis. Due to the large uncertainty at several nodes, members of a polytomy were profile aligned to find the best order. [file 1471-2148-9-37-S2.pdf]
